# Biosynthesis and Thermal Properties of PHBV Produced from Levulinic Acid by *Ralstonia eutropha*

**DOI:** 10.1371/journal.pone.0060318

**Published:** 2013-04-04

**Authors:** Yuanpeng Wang, Ronghui Chen, JiYuan Cai, Zhenggui Liu, Yanmei Zheng, Haitao Wang, Qingbiao Li, Ning He

**Affiliations:** Department of Chemical and Biochemical Engineering, College of Chemistry and Chemical Engineering, and The Key Laboratory for Synthetic Biotechnology of Xiamen City, Xiamen University, Xiamen, China; Dowling College, United States of America

## Abstract

Levulinic acid (LA) can be cost-effectively produced from a vast array of renewable carbohydrate-containing biomaterials. LA could facilitate the commercialization of the polymer poly(hydroxybutyrate-co-hydroxyvalerate) (PHBV) and PHBV-based products as carbon substrates. Therefore, this paper focused on the production of PHBV by *Ralstonia eutropha* with LA for hydroxyvalerate (HV) production, which plays an important role in enhancing the thermal properties of PHBV. Accordingly, the HV content of PHBV varied from 0–40.9% at different concentrations of LA. Stimulation of cell growth and PHBV accumulation were observed when 2–6 g L^−1^ LA was supplied to the culture. The optimal nitrogen sources were determined to be 0.5 g L^−1^ ammonium chloride and 2 g L^−1^ casein peptone. It was determined that the optimal pH for cell growth and PHBV accumulation was 7.0. When the cultivation was performed in large scale (2 L fermenter) with a low DO concentration of 30% and a pH of 7.0, a high maximum dry cell weight of 15.53 g L^−1^ with a PHBV concentration of 12.61 g L^−1^ (53.9% HV), up to 81.2% of the dry cell weight, was obtained. The melting point of PHBV found to be decreased as the fraction of HV present in the polymer increased, which resulted in an improvement in the ductility and flexibility of the polymer. The results of this study will improve the understanding of the PHBV accumulation and production by *R. eutropha* and will be valuable for the industrial production of biosynthesized polymers.

## Introduction

Biosynthesized polyhydroxyalkanoate (PHA) polymers are currently attracting much interest from researchers because of their physical properties, which are similar to those of conventional thermoplastics, such as polyethylene (PE) and polypropylene (PP) [1]–[3]. A variety of bacteria are known to use various carbon substrates to synthesize PHAs under limiting growth conditions [4]–[9], including *Ralstonia eutropha*, a model bacterium for PHA synthesis [10]–[11].

PHB is the first PHA that was identified and is most likely the best-characterized PHA. However, the degradation temperature of PHB is just a few degrees above its melting temperature, which results in instability during the melting stage. Additionally, the brittleness, hardness and crystalline nature of PHB limit its applications [12]. The incorporation of other monomeric units into HB polymer chains can lead to copolymers with improved properties [13]–[14]. Work focused on improving the properties of PHB has led to the copolymer of HB and 3-hydroxyvalerate (HV), namely poly(3-hydroxybutyrate/3-hydroxyvalerate) (PHBV) [15]. When compared to PHB homopolymer, the PHBV copolymer has better physical properties such as impact resistance, toughness, flexibility and other properties involved in the manufacturing process. Additionally, the performance of PHBV can vary greatly when this polymer contains different proportions of the HV monomer. Increasing the HV content in PHBV from 0 to 50% can significantly lower the melting point of the resulting polymer.

The structure of PHBV can be manipulated by the types of carbon sources supplemented into the medium. Dionisi et al. obtained a homopolymer of polyhydroxyvalerate (PHV) from propionate and a copolymer (34% HB and 66% HV) from a mixture of acetate and propionate [16]. Beccari et al. obtained a copolymer of HB and HV (50% HB and 50% HV) from a mixture of acetate, propionate, butyrate and valerate [17]. Several odd-numbered carbon sources (e.g., propionate and valerate) were supplemented into the medium to incorporate HV units into PHBV.

Although PHBV could be produced by different types of carbon sources, the main obstacle hindering the economical production of PHBV is the cost of the carbon substrate, which accounts for 28–50% of the total production cost during microbial fermentation [18]–[19]. Therefore, it is important to manipulate the substrate composition so that synthetic PHBV production by microbial fermentation is inexpensive. Levulinic acid (LA) is a renewable co-product that has drawn interest as substrates for PHA biopolymer synthesis. LA can be produced cost-effectively from a vast array of carbohydrate-containing renewable biomaterials, including cellulose-containing forest and agricultural waste residues, paper mill sludge, and cellulose fines from paper production processes. Because of this, economic projections indicate that LA production costs could fall to as low as $0.04–$0.10/lb depending on the scale of operation [20]–[22]. Several studies have demonstrated the use of LA as a sole carbon source or a co-substrate for cell growth and PHA biosynthesis [23]–[24]. LA can serve as a cheap alternative to conventional fermentation substrates. However, problems remain in using LA for PHA biosynthesis. A maximal PHA content of only 38.3% (w/w) was achieved when *Alcaligenes* sp. SH-69 was cultivated on glucose and LA [25]. Shaking-flask fermentation of *Burkholderia cepacia* (formerly *Pseudomonas cepacia*) containing 2.2% (w/v) xylose and concentrations of LA ranging from 0.07% to 0.67% (w/v) yielded 4.4–5.3 g L^−1^ of dry cell biomass, containing 42–56% (w/w) PHBV [26]. The low content of PHBV in biomass leads to a high cost for both production processes and downstream processing and thus restricts the use of LA for PHBV biosynthesis.

In this work, the effects of different constituents of the medium and other culture conditions for PHBV production by *R. eutropha* on LA during shaking-flask fermentation were investigated. Then, the optimal medium for PHBV production was applied in 2-L fermentation. Finally, the thermal properties of PHBV were analyzed using differential scanning calorimetry (DSC) and thermogravimetry (TG). The results of this study will improve the understanding of PHBV accumulation and production by microbes and be valuable for industrial polymer biosynthesis.

## Materials and Methods

### Bacterial Strain

*Ralstonia eutropha* H16 was used in all experiments. The strain was maintained on nutrient agar slants at 4°C and sub-cultured monthly.

### Media and Cultivation Conditions

Nutrient broth consisting of 10 g L^−1^ beef extract, 10 g L^−1^ tryptone and 2.0 g L^−1^ yeast extract was used for seed cultures. For the nutrient agar slants, 2% agar was added to this medium. The fermentation medium (MSM) contained 3.0 g L^−1^ Na_2_HPO_4_·12H_2_O, 0.5 g L^−1^ KH_2_PO_4_, 0.5 g L^−1^ NH_4_Cl, 0.1 g L^−1^ MgSO_4_·7H_2_O, 1.2 mg L^−1^ Fe(III)NH_4_-citrate and 10 mL L^−1^ trace element solution. The trace element solution consisted of 10 mg L^−1^ ZnSO_4_·7H_2_O, 3 mg L^−1^ MnCl_2_·4H_2_O, 30 mg L^−1^ H_3_BO_3_, 20 mg L^−1^ CoCl_2_·6H_2_O, 1 mg L^−1^ CuCl_2_·6H_2_O, 2 mg L^−1^ NiCl_2_·6H_2_O and 3 mg L^−1^ NaMoO_4_·2H_2_O. The initial pH of all media was adjusted to 7.0 (unless otherwise indicated) with NaOH (1 M) and HCl (0.5 M). The effects of using different LA co-carbon sources, including 20 g L^−1^ fructose, glucose, sucrose, lactose, dextrin, soluble starch, and molasses, on PHBV production were investigated. A series of nitrogen sources was also investigated. All media were prepared with distilled water and sterilized at 121°C for 30 min.

### Shaking-flask Fermentation

Overnight culture (16h) of *R. eutropha* was prepared using nutrient broth medium for inoculum. 50 mL of the same media in 250 mL flask were used for ferment of PHBV with 2.5 mL (5%, v/v) of inoculum under shaking conditions for 72 h at 30°C and 200 rpm.

### 2-L Fermentation

Batch cultivation was also carried out in a 2-L fermenter (Applikon BioBundle, Holland) containing 1.2 L of fermentation medium (MSM) with 5% (v/v) inoculum at 30°C and aeration (500 rpm and 2 vvm air volume/culture volume/min). The pH was maintained at 7.0 by adding 1 M LA or 1 M NaOH.

### PHBV Quantification

PHBV quantification was carried out according to the propanolysis method proposed by Riis and Mai [27], and Chen and Li [28] with modifications. Sealed tubes containing 2 mL of dichloroethane, 1.6 mL of propanol, 0.4 mL of hydrochloric acid, 200 µL of a propyl benzoate solution (internal standard) and the lyophilized bacterial pellets recovered from fermented solids were heated at 100°C for 4–6 h. After cooling to room temperature, 4 mL of distilled water was added for extraction. Thereafter, 0.6 µL of the organic phase was injected into a gas chromatograph at 220°C, which was equipped with a flame ionization detector (FID) and a SE-30 capillary column (30 m×0.25 mm×0.5 µm). N_2_ was used as the carrier gas. The temperature of the oven was programmed for the efficient separation of peaks. First, the oven temperature was held constant at 100°C for 1 min. Then, the temperature was increased to 170°C at a rate of 10°C/min, and this temperature was maintained for 5 min. The detector temperature was then increased to 250°C. Calibrations for PHB and PHV were performed with a standard of poly(3-hydroxybutyric-co-hydroxyvaleric acid) (12 wt. % PHV) of natural origin (Sigma-Aldrich Chem.).

### Thermal Properties of PHBV

The thermal properties of PHBV were analyzed by DSC (DSC204, Netzsch) and TG (TG209F1, Netzsch). Specimens weighing approximately 3 mg were used for the DSC study. Heating and cooling rates were maintained at 10°C/min during the DSC runs. The specimens were heated from −30 to 200°C for 3 min. The melting temperature (Tm), melting enthalpy (▵H_f_), and crystallinity (Xc) were obtained from the thermograms. Specimens weighing approximately 4 mg were used for the thermogravimetry (TG) study. The initial thermal decomposition temperature (T_eoi_), onset temperature (T_em_), finished thermal decomposition temperature (T_eof_), and thermal weight loss (Wt-loss) were obtained. The specimens were heated from 0 to 400°C at a rate of 10°C/min during the TG runs in order to test the temperature of thermal degradation.

### Statistical Analysis

All data were analyzed using Microsoft Excel, Origin and SPSS. The treatment effects were carried out with one-way ANOVA and the LSD multiple range test was used to determine the statistical significance (*P*<0.05) between pairs with SPSS.

## Results

### Growth of *R. eutropha* and PHBV Yield with different Carbon Sources

There was no significant difference in DCW and PHBV production from *R. eutropha* with increasing inoculum size (Fig. S1). So 5% (v/v) of inoculum was used in following shaking flask and fermenter experiments.

Although *R. eutropha* could grow when LA was used as the sole carbon source, the production of dry cell weight (DCW) and PHBV were lower (1.29 and 0.03 g L^−1^, respectively) than that in the presence of additional carbon source (4.39 and 3.18 g L^−1^, respectively) (Table 1). However, only PHB accumulated when glucose was used as sole carbon source, which indicated the possibility of use of LA as a precursor for PHV production. Even though, sucrose, lactose, dextrin, soluble starch, and molasses supported the growth of *R. eutropha,* but insignificant PHBV accumulation were observed. Thus, in the present study, *R. eutropha* produced significantly higher amounts of PHBV from LA in the presence of glucose as a co-substrate.

**Table 1:** Dry cell weight (DCW) and PHBV obtained on different carbon sources in flask fermentation.

Carbon sources	DCW(g L^−1^)	PHBV(g L^−1^)	HV content (%)
LA^a^	1.29±0.05	0.03±0.01	52.7±4.6
Glucose+LA	4.39±0.44	3.18±0.25	21.4±1.7
Maltose+LA	0.00±0.00	–	–
Sucrose+LA	1.86±0.12	0.09±0.02	18.6±2.1
Lactose+LA	1.98±0.16	0.39±0.04	22.5±1.3
Fructose+LA	0.00±0.00	–	–
Dextrin+LA	2.01±0.47	0.03±0.01	15.9±1.6
Soluble starch+LA	1.80±0.32	0.05±0.05	16.3±1.1
Molasses+LA	2.68±0.54	0.66±0.27	21.9±2.8
Glucose (no LA)^b^	3.90±0.39	2.44±0.59	0

50 mL (pH 7.0) of media was in 250 mL flask with 5% (v/v) inoculum under shaking conditions at 200 rpm for 72 h at 30°C.

a 5 g L^−1^ LA was added as sole carbon source.

b 20 g L^−1^ glucose was added as sole carbon source.

### Effect of different Concentrations of Glucose and LA on PHBV Accumulation by *R. eutropha*

**Figure 1:**
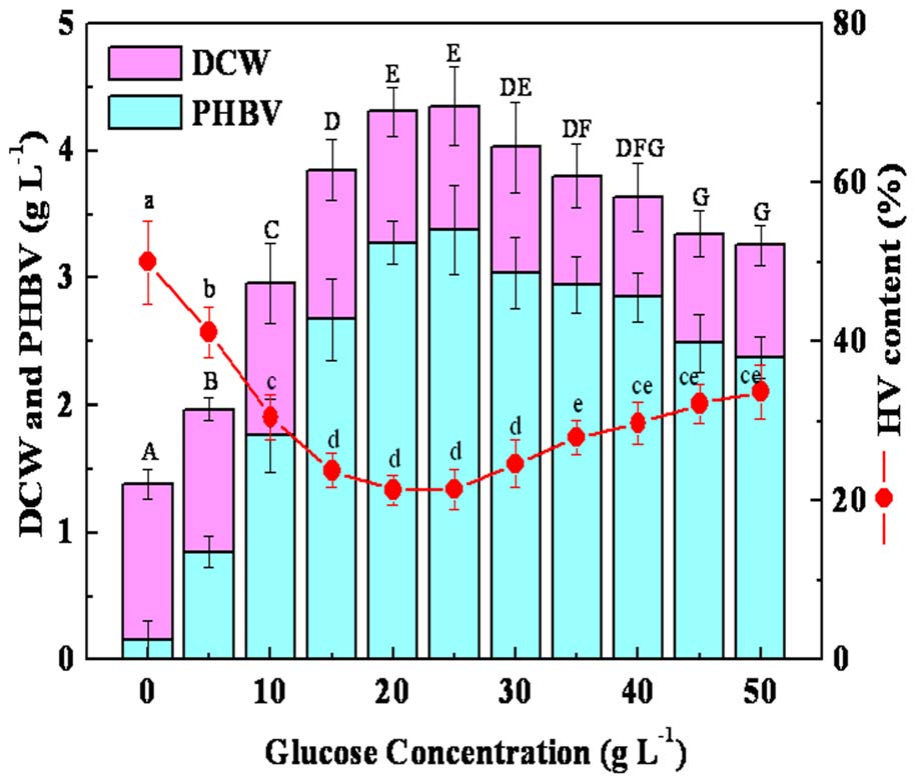
Effect of different glucose concentrations on DCW, PHBV and HV production. Sharing a common lowercase are not significantly different in the HV content and the same capital are not significantly different in the concentration of DCW and PHBV (*P*<0.05). 50 mL (pH 7.0) of media was in 250 mL flask with 5% (v/v) inoculum under shaking conditions at 200 rpm for 72 h at 30°C.

According to the experimental data with different concentration of glucose as a co-substrate in the presence of constant amount of LA (5 g L^−1^), DCW and PHBV concentration showed increasing trend with concentration of glucose up to 25 g L^−1^ followed by progressive fall of production. In contrast, concentration of HV decreased with an increasing concentration of glucose (0–25 g L^−1^) and increased with an increasing concentration of glucose (25–35 g L^−1^). However, the concentration of HV remained nearly constant at glucose concentrations greater than 35 g L^−1^ (Fig. 1).

**Figure 2:**
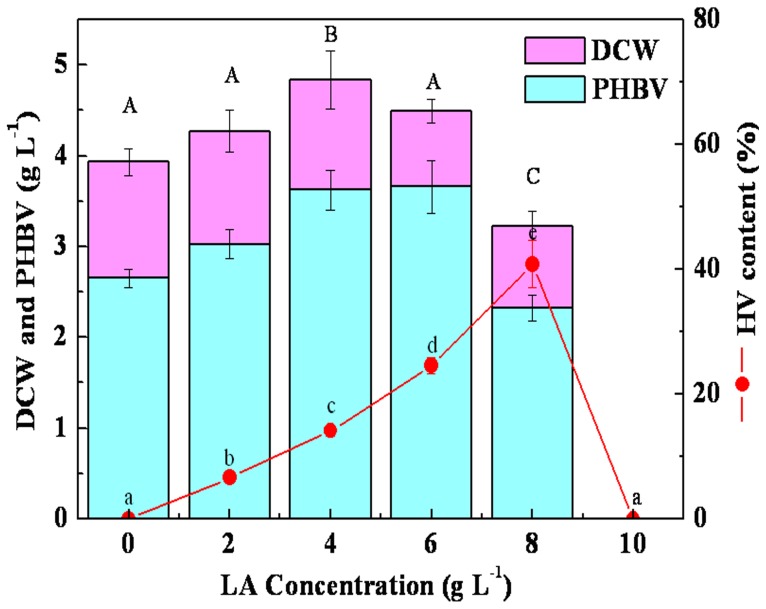
Effect of different LA concentrations on DCW, PHBV and HV production. Sharing a common lowercase are not significantly different in the HV content and the same capital are not significantly different in the concentration of DCW and PHBV (*P*<0.05). 50 mL (pH 7.0) of media was in 250 mL flask with 5% (v/v) inoculum under shaking conditions at 200 rpm for 72 h at 30°C.

DCW and PHBV were also affected by the amount of LA in the medium. Increment of DCW and PHBV production were observed during the raise of LA concentration up to 6 g L^−1^ beyond which showed significant reduction and complete inhibition of DCW and PHBV with 10 g L^−1^ LA. (Fig. 2).

### Effects of Nitrogen Sources on PHBV Accumulation by *R. eutropha*

Production of PHBV by *R. eutropha* was found to be significantly affected by the nitrogen concentration. Accordingly, there was a significant increase in DCW after the addition of ammonium chloride, ammonium sulfate and urea as a source of inorganic nitrogen, however, a higher PHBV production observed with the addition of ammonium chloride compared to other inorganic nitrogen source used (Table S1). Similar experimental evidences also reviled that casein peptone found to be better source of organic nitrogen source. Supporting the fact that, a maximum DCW (5.74 g L^−1^) and PHBV (3.86 g L^−1^) were obtained with the combination of those two organic and inorganic nitrogen sources (Table S2).

**Figure 3:**
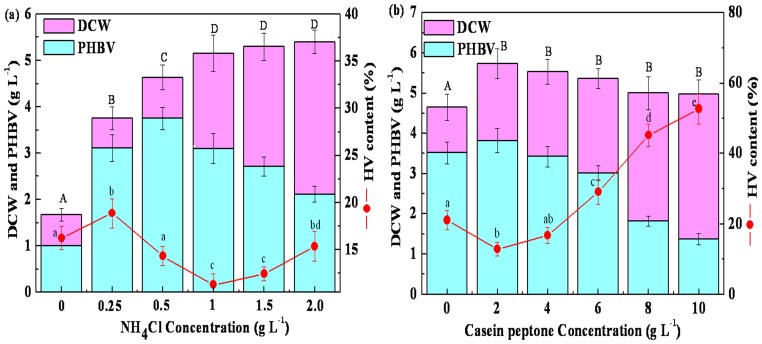
Effects of NH_4_Cl (a) and casein peptone (b) concentration on DCW, PHBV and HV production. Sharing a common lowercase are not significantly different in the HV content and the same capital are not significantly different in the concentration of DCW and PHBV (*P*<0.05). 50 mL (pH 7.0) of media was in 250 mL flask with 5% (v/v) inoculum under shaking conditions at 200 rpm for 72 h at 30°C.

According to our experimental data revealed in Fig. 3a, cell growth showed increasing with the concentration of NH_4_Cl, whereas the PHBV production increased up to 0.5 g L^−1^ and then progressively decreased. Thus, the optimized NH_4_Cl concentration was found to be 0.5 g L^−1^. Likewise, the cell growth and PHBV production with different concentrations of casein peptone were also investigated. As shown in Fig. 3b, PHBV production decreased with an increase in casein peptone concentration while the cell growth remained almost the same. Thus, the optimized casein peptone concentration was found to be 2.0 g L^−1^.

### Effects of the Initial pH on PHBV Accumulation by *R. eutropha*

**Figure 4:**
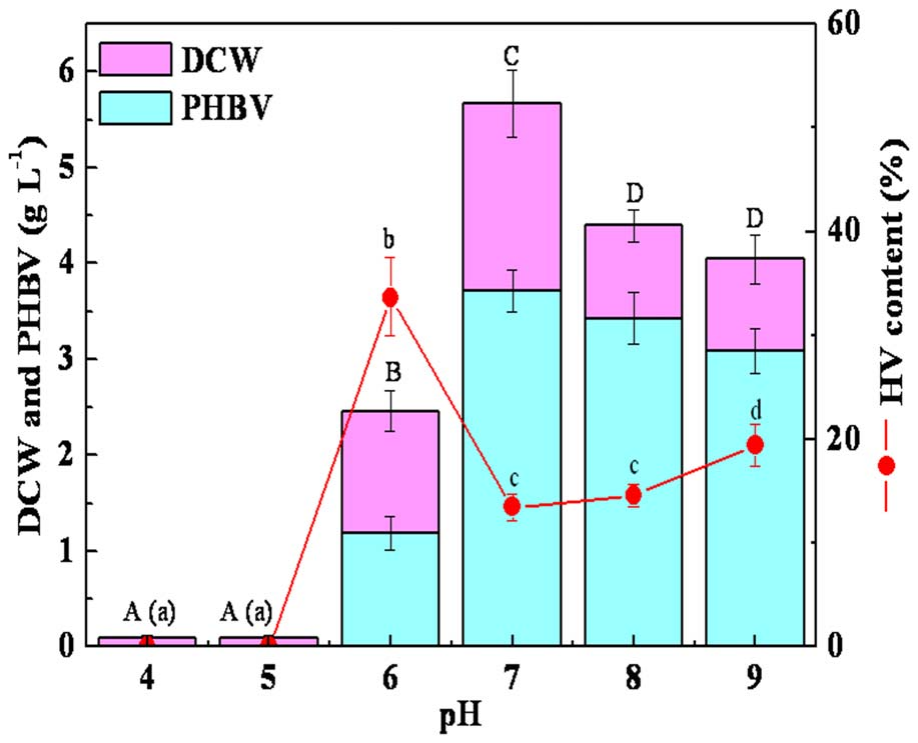
Effects of initial pH on DCW, PHBV and HV production. Sharing a common lowercase are not significantly different in the HV content and the same capital are not significantly different in the concentration of DCW and PHBV(*P*<0.05). 50 mL of media was in 250 mL flask with 5% (v/v) inoculum under shaking conditions at 200 rpm for 72 h at 30°C.

Cell growth was completely inhibited under pH 5.0. However, a maximum DCW of 5.67 g L^−1^ and PHBV concentration of 3.71 g L^−1^ was obtained when the pH was 7.0 (Fig. 4). Although the final pH of the culture broth ranged from 8.20∼8.90 throughout all experiments, an initial pH of 8.0 or 9.0 resulted in decreased PHBV accumulation by *R. eutropha* compared to that of cultures with an initial pH of 7.0.

### Accumulation of PHBV in a 2-L Fermenter

**Figure 5:**
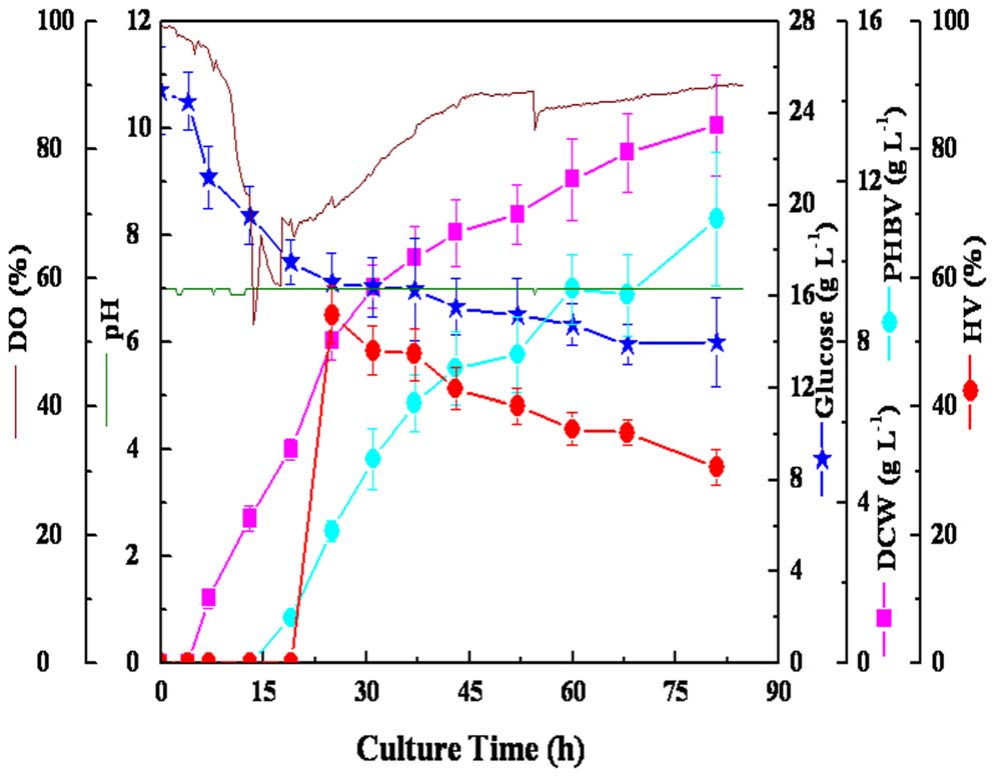
Batch cultivation of *R. eutropha* for PHBV accumulation under the condition of stable pH, which was automatically fed to keep the pH of the culture at 7.0. 1.2 L (pH 7.0) of media was in 2 L fermenter with 5% (v/v) inoculum at 30°C and aeration/agitation (500 rpm and 2 vvm air volume/culture volume/min).

The 250 mL flask experiment proved that the pH value is an important parameter for PHBV production and that PHBV production can be significantly improved by controlling the pH. Therefore, 25 g L^−1^ glucose and 4 g L^−1^ LA were added at the beginning as the carbon sources in a 2-L fermenter, and the culture was automatically fed to keep the pH at 7.0. Fig. 5 shows the time course of *R. eutropha* growth in a 2-liter fermenter at pH 7.0. At 16 h, there was a significant decrease in the concentration of DO, which was followed by an increase in DO to a concentration of 90%. The dry cell weight increased to 13.41 g L^−1^ at 81 h. PHBV reached a final concentration of 11.08 g L^−1^, up to 82.6% of the cell weight. However, the HV content was only 30.6% (w/w).

**Figure 6:**
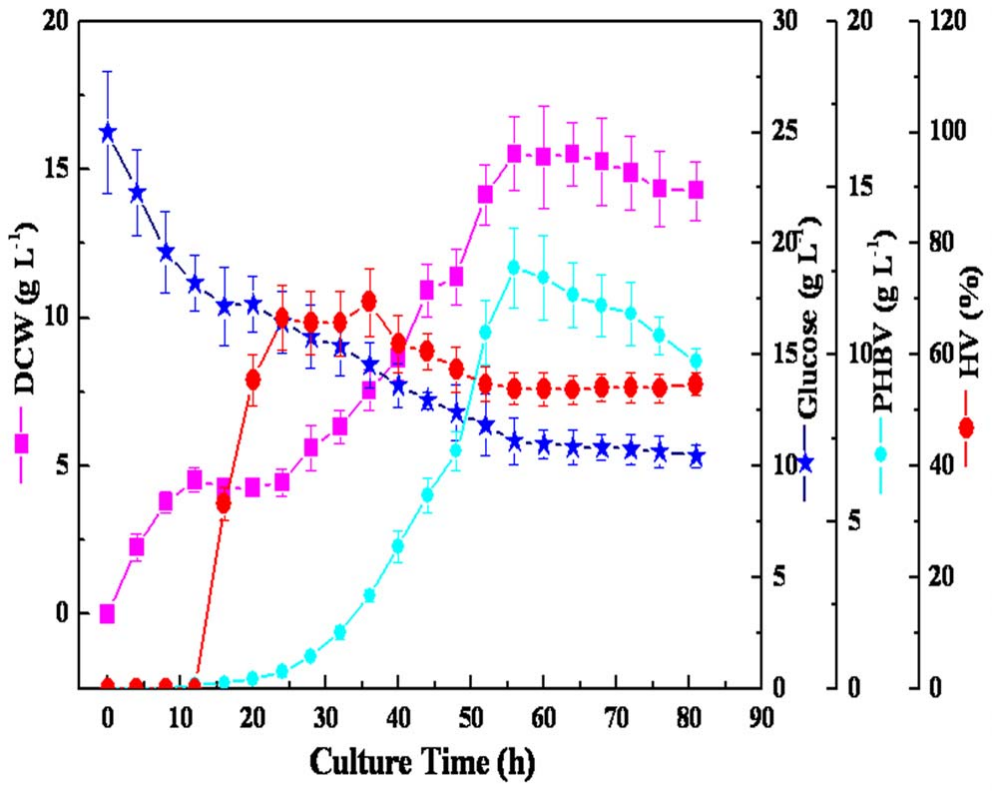
Batch cultivation of *R. eutropha* for PHBV accumulation under the condition of stable pH and DO, which was automatically kept at 7.0 and 30%, respectively. 1.2 L (pH 7.0) of media was in 2 L fermenter with 5% (v/v) inoculum at 30°C and aeration.

To enhance the HV content in PHBV, the 2-L fermenter was programmed to automatically keep the DO of the culture at 30% at pH 7.0 (Fig. S2). Aliquots of 25 g L^−1^ glucose and 4 g L^−1^ LA were also added as carbon sources. Fig. 6 shows the growth curve of *R. eutropha* in a 2-Lfermenter. The dry cell weight increased to 15.53 g L^−1^ in 56 h, and PHBV reached a final concentration of 12.61 g L^−1^, up to 81.2% of the cell weight. The highest HV content obtained was 53.9% (w/w).

### HV Fractions and Thermal Properties

**Figure 7:**
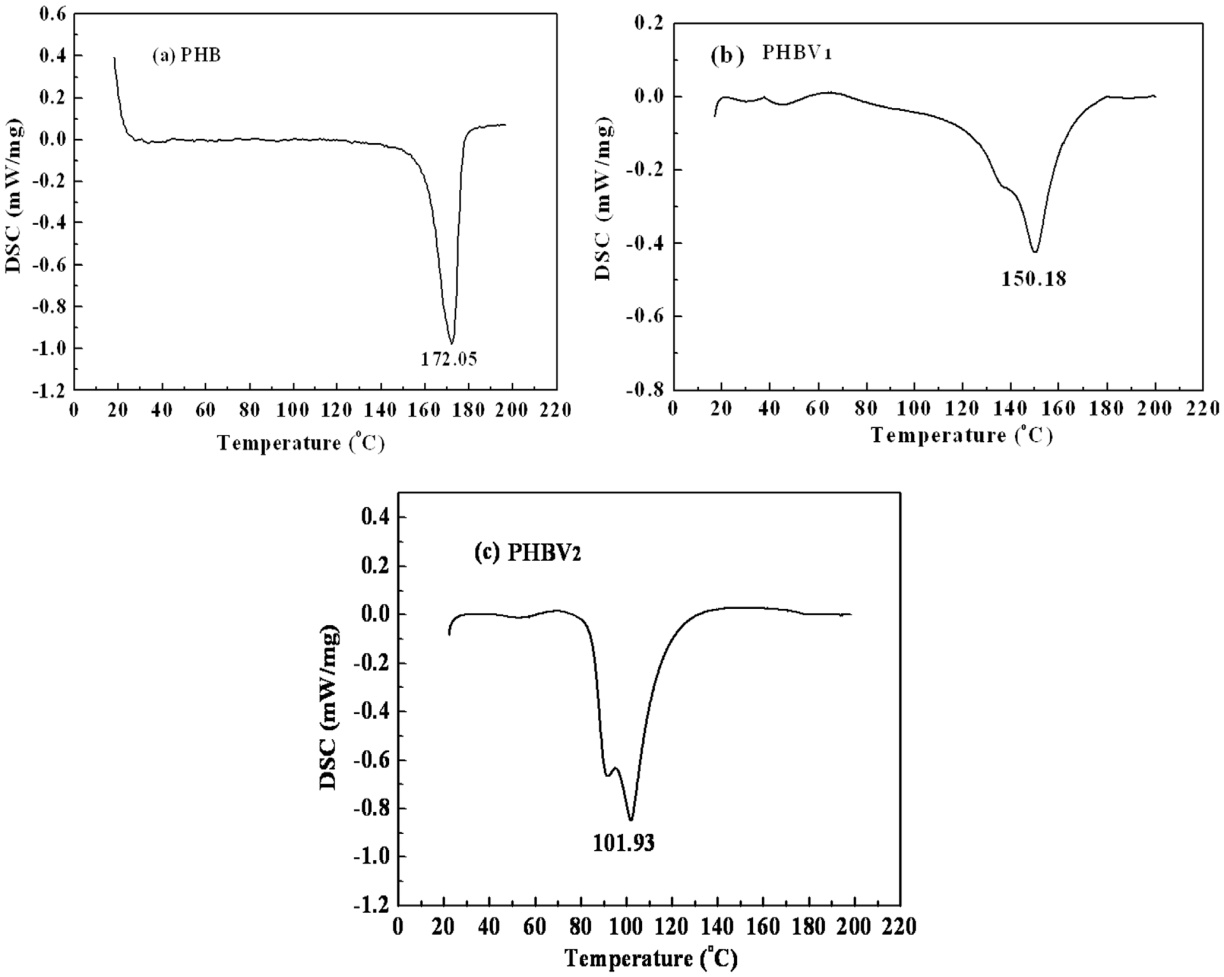
DSC thermograms of PHB (a), PHBV_1_ (b) and PHBV_2_ (c) samples.

The thermal properties of PHBV (containing 0, 16 and 53% of HV monomer, respectively) were analyzed by DSC and TG. Fig. 7 shows the DSC thermograms of the different PHBV specimens. The degree of crystallinity (Xc), melting point (Tm), and melting enthalpy (▵H_f_) of the PHBV specimens are summarized in Table 2. The data in Table 2 suggest that the degree of crystallinity of PHBV increased with decreasing HV content in the polymer, which might mean that the three polymers exhibited obvious differences in crystallinity. The melting point of PHBV also decreased with an increase in the amount of HV, which potentially resulted in an improvement in the ductility and flexibility of the polymer.

**Table 2:** Thermal properties of PHBV samples with different fractions of HV.

	DSC				TG			
Sample	HV	X_C_	T_m_	▵H_f_	T_eoi_	T_em_	T_eof_	Wt-Loss
PHB	0	61.44	172.05	89.7	249.6	263.4	269.8	88.72
PHBV_1_	16%	50.34	150.18	73.5	266.6	298.3	315.9	85.60
PHBV_2_	53%	51.92	101.93	75.8	252.3	284.4	303.6	85.87

Xc: crystallinity, Tm: melting point, ▵H_f_: melting enthalpy, T_eoi_: initial thermal decomposition temperature, T_em_: onset temperature, T_eof_: finished thermal decomposition temperature, Wt-loss: thermal weight loss.

**Figure 8:**
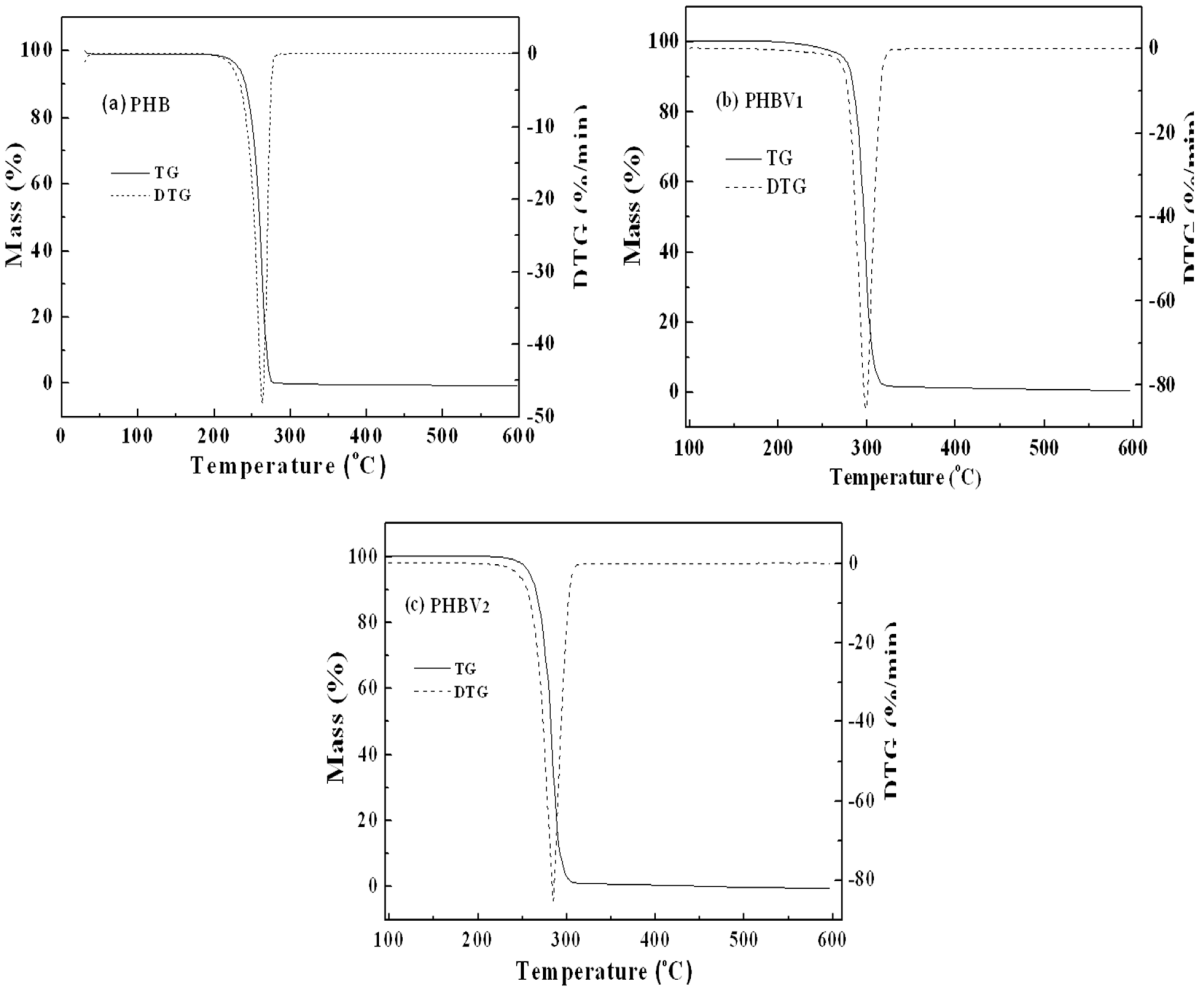
TG-DTG curve of PHB (a), PHBV1 (b) and PHBV2 (c) samples.

Fig. 8 shows the TG graph of the different PHBV specimens. The results of a TG graph analysis are also listed in Table 2. It can be seen that the thermal decomposition temperatures of three PHBV specimens were in the range of 240–290°C, and there were no significant decreases in T_eoi_, T_em_, T_eof_ with increasing HV content.

## Discussion

Recent studies have shown that PHB is commonly produced at an industrial level from *Cupriavidus necator*, *Alcaligenes latus* and *R. eutropha* [11], [29]–[30]. However, a melting temperature that is close to the degradation temperature and the brittle, hard and crystalline nature of PHB limits its applications [12]. In this study, the bacterium *R. eutropha* was able to use LA as the co-carbon source to synthesize the copolymer PHBV in shaking-flask and 2-L fermentations.

LA can be produced cost-effectively from a vast array of carbohydrate-containing renewable biomaterials. Because of this, economic projections indicate that LA production costs could fall to as low as $0.04–$0.10/lb depending on the scale of operation [20]–[22]. Since LA is cheap and is a structural analog of pentanoic acid, it has been assessed as a secondary substrate in PHA biosynthesis. In present study, the optimized PHBV yield in a 2-L fermenter was 12.61 g L^−1^, up to 81.2% of the cell weight when LA was the co-carbon source (Fig. 6). The PHBV content was much higher than that achieved by *Burkholderia* sp. IS-01 in a 7-L fermenter, which contained gluconate (20 g L^−1^) and LA (5 to 12.5 g L^−1^) as the co-substrates [31]. Additionally, although high concentration of LA is known to be toxic to microorganisms and to lead to a decrease in growth, there was a significant increase in the DCW of *R. eutropha* as the concentration of LA was increased from 2 to 8 g L^−1^ in this study (Fig. 2). This result was quite different from that obtained with *R. eutropha* KHB-8862, in which LA addition showed no stimulatory effect on cell growth [32]. Although a stimulatory effect of LA addition has been observed [25], it has been restricted to a relatively low concentration (0.5 g L^−1^) of LA. Conversely, the maximum cell growth and, consequently, the maximum PHBV production were highly dependent on the nitrogen source and the initial pH. Even though previous studies have described urea as the best nitrogen source for PHA production by *R. eutropha* [33]–[34], our experimental evidence reviled that using NH_4_Cl and casein peptone, 70% more PHBV was produced compared to the batch fermentation using urea as the initial nitrogen source (Fig. 3, Table S1).

Although LA can act as co-carbon source for PHBV production, no significant PHBV was accumulated when sucrose, lactose, dextrin, soluble starch, and molasses were used as carbon sources (Table 1). More importantly, when glucose was chosen as the sole carbon source, only PHB accumulated, which indicated that LA was the precursor for PHV and might be an effective means by which to control the HV content in the copolyesters. In this study, as the concentration of LA varied from 2 to 8 g L^−1^, the HV content significantly increased (Fig. 2). It was suggested that LA is first activated to form levulinyl-CoA and then split into propionyl-CoA and acetyl-CoA. The two intermediates are either used for cell growth via the main metabolism pathway or condensed into 3-ketovaleryl-CoA for HV via the well-established PHA biosynthesis pathway (Fig. S3). This might be the reason for the increase in the HV content when the LA concentration was increased because more propionyl-CoA was formed. It was also shown that the percentage of HV reached a maximum at 36 h before the most active PHBV biosynthesis occurred (Fig. 6). Thereafter, the percentage of HV declined while PHBV production increased. This may be due to the higher synthesis rate of HB because the synthesis of HV depends on the level of metabolism needed to convert the precursor substrates into their corresponding hydroxyacyl-CoA thioesters [35].

In addition to the initial LA concentration, the concentrations of the nitrogen source and DO also have important roles in determining the HV content of PHBV. There was a significant increase in the HV content with an increasing concentration of casein peptone (Fig. 3). It has been proposed that HV production was significantly influenced by suitable C:N ratio [28], [31]. More importantly, 54% HV content could be produced at a low concentration of DO; however, only 30.6% HV content was synthesized at a high concentration of DO (Figs. 5 and 6). A previous study also demonstrated that HV was easily accumulated by microorganisms under such unbalanced growth conditions of DO that combined intermittent aeration and nutrient limitation [2]. Accordingly, obtaining the desired fraction of HV in PHBV requires the proper ration of C:N and stress resistance conditions.

The fraction of HV present in PHBV had a significant effect on the melting point, which decreased with an increase in the amount of HV in PHBV. This resulted in an improvement in the ductility and flexibility of the polymer. In this study, three PHBV specimens did not begin thermal decomposition until 220°C (Fig. 8 and Table 2), which is in agreement with a previous report [14], [36]. Because the melting temperatures of three PHBV specimens were approximately 100–150°C and their thermal decomposition temperatures were above 220°C, it is clear that increasing the HV content in the PHBV polymer greatly improved its workability.

To conclude, biodegradable polymers, especially PHBV, will certainly play an important role in the plastics market in the future due to their biodegradability and to the use of renewable resources for their production. Although their manufacturing costs today are still too high to compete with conventional petroleum-based polymers, LA has great potential for large-scale production of the polymer as it can be produced cost-effectively. When batch cultivation was conducted in a 2-L lab scale fermenter, a significantly higher maximum dry cell weight of 15.53 g L^−1^ with a PHBV concentration of 12.61 g L^−1^ (53.9% HV), up to 81.2% of the dry cell weight, was obtained. More importantly, a desired fraction of HV in the PHBV could be obtained with the proper C:N ratio and stress resistance conditions, which will certainly improve PHBV competitiveness and make the broad use of these biopolymers possible in the future.

## Supporting Information

Figure S1 **Effect of inoculum size on cell growth and PHBV production from** ***R. eutropha.***
(DOC)

Figure S2 **Online data of batch cultivation under DO-stat control along with pH-stat control.**
(DOC)

Figure S3 **Metabolic pathway of PHBV synthesis from levulinic acid (CoA, coenzyme-A; ATP, adenosine triphosphate; PhaA, β-ketothiolase A; BktB, β-ketothiolase B; PhaB, NADPH-dependent acetoacetyl-CoA reductase; NADPH, nicotinamide adenine dinucleotide phosphate; PhaC, PHA synthase).**
(DOC)

Table S1 **Dry cell weight (DCW) and PHBV obtained on different nitrogen sources.**
(DOC)

Table S2 **Dry cell weight (DCW) and PHBV obtained on different organic nitrogen sources with or without ammonium chloride.**
(DOC)

Text S1 (DOC)
