# Supplementary material for: Biosynthesis and Thermal Properties of PHBV Produced from Levulinic Acid by Ralstonia eutropha
Source: PLoS One. 2013 Apr 4;8(4):e60318. doi: 10.1371/journal.pone.0060318 (PMC3617235; doi:10.1371/journal.pone.0060318)
Supplement: Figure S1 — Effect of inoculum size on cell growth and PHBV production from R. eutropha. (DOC) [file pone.0060318.s001.doc]

**Fig. S1** Effect of inoculum size on cell growth and PHBV production from *R. eutropha*
