# Supplementary material for: Biosynthesis and Thermal Properties of PHBV Produced from Levulinic Acid by Ralstonia eutropha
Source: PLoS One. 2013 Apr 4;8(4):e60318. doi: 10.1371/journal.pone.0060318 (PMC3617235; doi:10.1371/journal.pone.0060318)
Supplement: Figure S2 — Online data of batch cultivation under DO-stat control along with pH-stat control. (DOC) [file pone.0060318.s002.doc]

**Fig. S2** Online data of batch cultivation under DO-stat control along with pH-stat control
