# Supplementary material for: Biosynthesis and Thermal Properties of PHBV Produced from Levulinic Acid by Ralstonia eutropha
Source: PLoS One. 2013 Apr 4;8(4):e60318. doi: 10.1371/journal.pone.0060318 (PMC3617235; doi:10.1371/journal.pone.0060318)
Supplement: Figure S3 — Metabolic pathway of PHBV synthesis from levulinic acid (CoA, coenzyme-A; ATP, adenosine triphosphate; PhaA, β-ketothiolase A; BktB, β-ketothiolase B; PhaB, NADPH-dependent acetoacetyl-CoA reductase; NADPH, nicotinamide adenine dinucleotide phosphate; PhaC, PHA synthase). (DOC) [file pone.0060318.s003.doc]

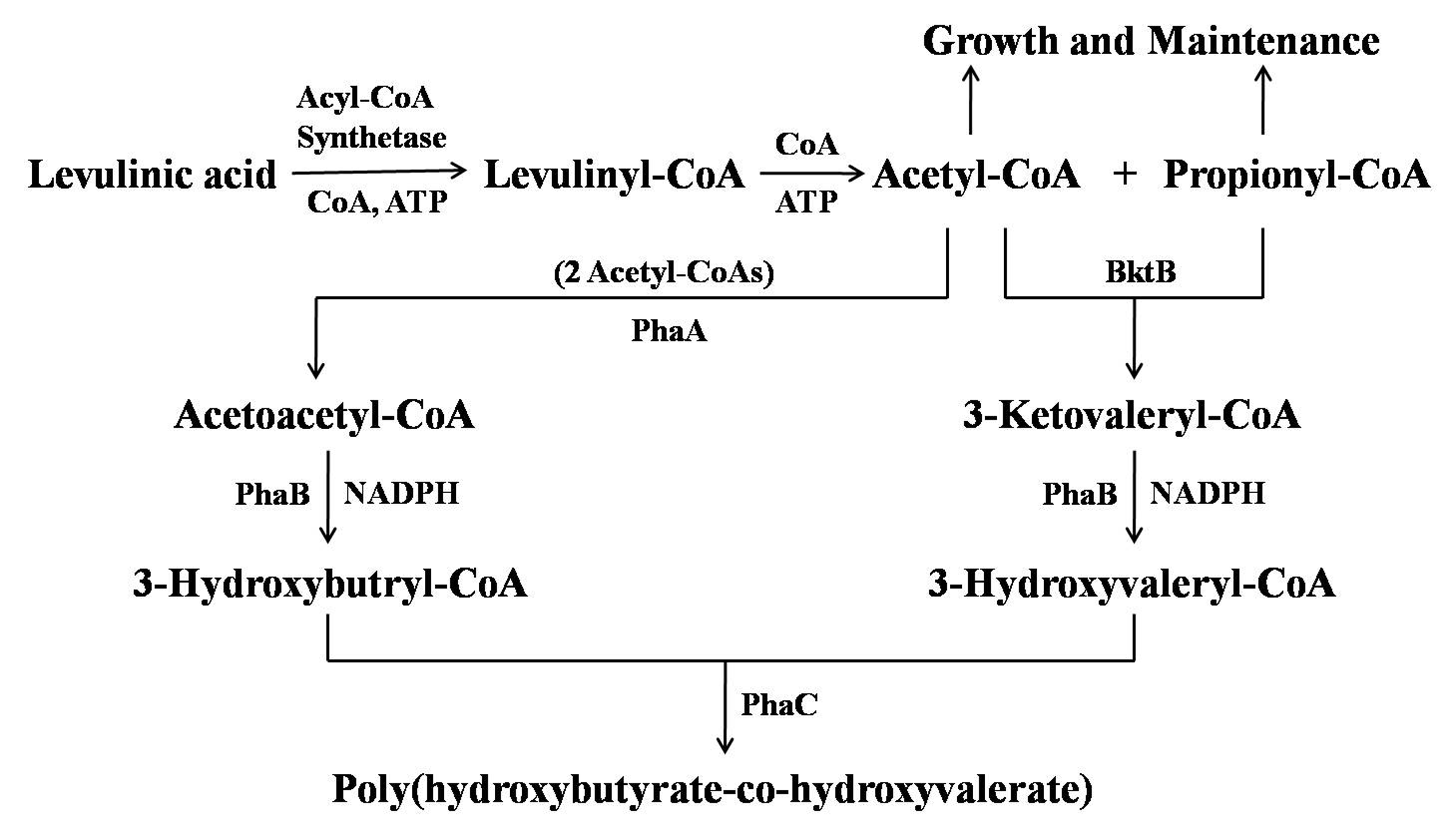


**Fig. S3** Metabolic pathway of PHBV synthesis from levulinic acid (CoA, coenzyme-A; ATP, adenosine triphosphate; PhaA, β-ketothiolase A; BktB, β-ketothiolase B; PhaB, NADPH-dependent acetoacetyl-CoA reductase; NADPH, nicotinamide adenine dinucleotide phosphate; PhaC, PHA synthase) [1]
