# Supplementary material for: Biosynthesis and Thermal Properties of PHBV Produced from Levulinic Acid by Ralstonia eutropha
Source: PLoS One. 2013 Apr 4;8(4):e60318. doi: 10.1371/journal.pone.0060318 (PMC3617235; doi:10.1371/journal.pone.0060318)
Supplement: Table S1 — Dry cell weight (DCW) and PHBV obtained on different nitrogen sources. (DOC) [file pone.0060318.s004.doc]

**Table S1.** Dry cell weight (DCW) and PHBV obtained on different nitrogen sources

| Nitrogen sources | DCW(g L^-1^) | PHBV(g L^-1^) |
| --- | --- | --- |
| Control^a^ | 1.67±0.13 | 1.07±0.10 |
| Ammonium chloride | 4.65±0.36 | 3.56±0.28 |
| Ammonium sulfate | 4.69±0.29 | 2.54±0.17 |
| Urea | 4.92±0.39 | 2.14±0.14 |
| Yeast extract powder | 2.71±0.21 | 1.26±0.16 |
| Casein peptone | 2.56±0.19 | 1.46±0.15 |
| Sodium nitrate | 3.85±0.35 | 2.06±0.27 |

^a^ No nitrogen source was added to the fermentation medium
