# Supplementary material for: Biosynthesis and Thermal Properties of PHBV Produced from Levulinic Acid by Ralstonia eutropha
Source: PLoS One. 2013 Apr 4;8(4):e60318. doi: 10.1371/journal.pone.0060318 (PMC3617235; doi:10.1371/journal.pone.0060318)
Supplement: Table S2 — Dry cell weight (DCW) and PHBV obtained on different organic nitrogen sources with or without ammonium chloride. (DOC) [file pone.0060318.s005.doc]

**Table S2.** Dry cell weight (DCW) and PHBV obtained on different organic nitrogen sources with or without ammonium chloride

| Carbon sources | DCW(g L^-1^) | PHBV(g L^-1^) |
| --- | --- | --- |
| Yeast extract powder | 5.13±0.41 | 3.34±0.29 |
| Yeast extract powder & Ammonium chloride | 5.46±0.37 | 3.35±0.28 |
| Tryptone | 2.98±0.24 | 2.11±0.19 |
| Tryptone & Ammonium chloride | 4.85±0.37 | 3.48±0.29 |
| Beef extract | 3.25±0.22 | 1.89±0.14 |
| Beef extract & Ammonium chloride | 5.08±0.29 | 3.58±0.30 |
| Casein peptone | 4.14±0.27 | 2.90±0.19 |
| Casein peptone & Ammonium chloride | 5.74±0.42 | 3.82±0.33 |
| Soya peptone | 2.82±0.19 | 1.88±0.13 |
| Soya peptone & Ammonium chloride | 5.73±0.47 | 3.86±0.32 |
