# Supplementary material for: Biosynthesis and Thermal Properties of PHBV Produced from Levulinic Acid by Ralstonia eutropha
Source: PLoS One. 2013 Apr 4;8(4):e60318. doi: 10.1371/journal.pone.0060318 (PMC3617235; doi:10.1371/journal.pone.0060318)
Supplement: Text S1 — (DOC) [file pone.0060318.s006.doc]

**Biosynthesis and thermal properties of PHBV Produced from levulinic acid by *Ralstonia eutropha***

Yuanpeng Wang, Ronghui Chen, Jiyuan Cai, Zhenggui Liu, Yanmei Zheng, Haitao Wang, Qingbiao Li and Ning He[[1]](#footnote-2)

Department of Chemical and Biochemical Engineering, College of Chemistry and Chemical Engineering, and The Key Laboratory for Synthetic Biotechnology of Xiamen City, Xiamen University, Xiamen 361005, China

**Effect different nitrogen sources on DCW and PHBV production**

Different nitrogen sources were investigated in present study. Table S1 showed the DCW and PHBV obtained with 0.5 g L^-1^ relevant nitrogen source. There was significant increase in DCW after the addition of ammonium chloride, ammonium sulfate and urea. However, a higher PHBV production was accumulated by addition of ammonium chloride. Thus ammonium chloride was used subsequently in this work.

It was noticed that the dry cell weight was relatively low since the concentration of NH_4_Cl must be limited for PHBV production. 2 g L^-1^ relevant organic nitrogen was then added with or without 0.5 g L^-1^ NH_4_Cl as the nitrogen sources. As shown in Table S2, ammonium chloride turned out to be more effective than organic nitrogen as sole nitrogen source. Better cell growth was gained when organic nitrogen source was mixed with ammonium chloride and considerable PHBV production (3.35-3.86 g L^-1^) was gained compared to that on ammonium chloride (3.52 g L^-1^). Among the chosen organic nitrogen sources, yeast extract powder was the best as sole nitrogen, the dry cell weight and PHBV concentration were 5.13 g L^-1^ and 3.34 g L^-1^ respectively. However, casein peptone was more suitable when mixed with ammonium chloride. A maximum dry cell weight (5.74 g L^-1^) and PHBV (3.86 g L^-1^) were gained by compound nitrogen sources.

**Effect of inoculum size on DCW and PHBV production**

High inoculum size increases the HV production compared to low inoculum size. However, there was no significant difference in DCW and PHBV production from *R. eutropha* with increasing inoculum size (Fig. S1).

References

1. Jaremko M, Yu J. (2011) The initial metabolic conversion of levulinic acid in Cupriavidus necator. J Biotechnol 155: 293-298.

1. Corresponding author. Tel: +86 592 2183088; fax: +86 592 2184822.

   E-mail address: [hening@xmu.edu.cn](mailto:hening@xmu.edu.cn) [↑](#footnote-ref-2)
